# Trunk Muscle Mass Measured by Bioelectrical Impedance Analysis Reflecting the Cross-Sectional Area of the Paravertebral Muscles and Back Muscle Strength: A Cross-Sectional Analysis of a Prospective Cohort Study of Elderly Population

**DOI:** 10.3390/jcm10061187

**Published:** 2021-03-12

**Authors:** Hamidullah Salimi, Shoichiro Ohyama, Hidetomi Terai, Yusuke Hori, Shinji Takahashi, Masatoshi Hoshino, Akito Yabu, Hasibullah Habibi, Akio Kobayashi, Tadao Tsujio, Shiro Kotake, Hiroaki Nakamura

**Affiliations:** 1 Department of Orthopaedic Surgery, Osaka City University Graduate School of Medicine, Osaka 545-8585, Japan; hamidullahsalimi@yahoo.com (H.S.); ohyama.shoichiro@med.osaka-cu.ac.jp (S.O.); yusukehori0702@gmail.com (Y.H.); shinji@med.osaka-cu.ac.jp (S.T.); hirotoy@msic.med.osaka-cu.ac.jp (M.H.); yabuakito@gmail.com (A.Y.); drhasibhabibi@gmail.com (H.H.); hnakamura@med.osaka-cu.ac.jp (H.N.); 2 Department of Orthopaedic Surgery, Shiraniwa Hospital, Nara 630-0136, Japan; ak@med.osaka-cu.ac.jp (A.K.); t-tsujio@siren.ocn.ne.jp (T.T.); 3 Kotake Orthopaedic Clinic, Nara 631-0003, Japan; kotakeseikei@gmail.com

**Keywords:** trunk muscle, bioelectrical impedance analysis, MRI, back muscle strength

## Abstract

Trunk muscles play an important role in supporting the spinal column. A decline in trunk muscle mass, as measured by bioelectrical impedance analysis (TMM–BIA), is associated with low back pain and poor quality of life. The purpose of this study was to determine whether TMM–BIA correlates with quantitative and functional assessments traditionally used for the trunk muscles. We included 380 participants (aged ≥ 65 years; 152 males, 228 females) from the Shiraniwa Elderly Cohort (Shiraniwa) study, for whom the following data were available: TMM–BIA, lumbar magnetic resonance imaging (MRI), and back muscle strength (BMS). We measured the cross-sectional area (CSA) and fat-free CSA of the paravertebral muscles (PVM), including the erector spinae (ES), multifidus (MF), and psoas major (PM), on an axial lumbar MRI at L3/4. The correlation between TMM–BIA and the CSA of PVM, fat-free CSA of PVM, and BMS was investigated. TMM–BIA correlated with the CSA of total PVM and each individual PVM. A stronger correlation between TMM–BIA and fat-free CSA of PVM was observed. The TMM–BIA also strongly correlated with BMS. TMM–BIA is an easy and reliable way to evaluate the trunk muscle mass in a clinical setting.

## 1. Introduction

Trunk muscles, especially the paravertebral muscles (PVM), play an important role in supporting the spinal column [1]. The trunk muscles, which include the erector spinae (ES), multifidus (MF), and psoas major (PM), are reported to provide spinal stability during both moving and static states [2]. A decrease in trunk muscle volume or quality, due to sarcopenia [3] and fatty infiltration, along with aging, leads to spinal problems such as low back pain [4] and spinal sagittal imbalance [5]. Therefore, the importance of assessing trunk muscles, especially for the elderly in clinical settings, has attracted attention in recent years.

Traditionally, the quantitative assessment of trunk muscles is performed by measuring the cross-sectional area (CSA) of PVM using magnetic resonance imaging (MRI) or computed tomography (CT) [6,7,8,9], and the functional assessment of trunk muscles is performed by measuring back muscle strength (BMS) [10,11]. However, quantitative assessment of trunk muscles using MRI or CT is not routinely performed owing to the high cost and time requirements [12].

Bioelectrical impedance analysis (BIA) is a non-invasive examination technique that determines body composition by measuring the electrical resistance (bioimpedance) of living tissues [13]. In recent years, it has frequently been used in clinical settings as a guiding tool for fluid management and identification of the optimal method for patients undergoing dialysis [14,15,16]. BIA has been widely used to determine appendicular skeletal muscle mass (ASM) for diagnosis of sarcopenia [17]. In the limbs, which are mainly composed of muscle, bone, and fat, the muscle mass calculated by BIA is considered to be reliable [18].

Moreover, BIA has been used to calculate trunk muscle mass (TMM–BIA) and, recently, a decline in TMM–BIA has been associated with low back pain and poor quality of life [12]. However, it has been unclear whether the TMM–BIA reflects actual muscle mass, since the trunk also contains organs. Only one study [19] has reported a correlation between TMM–BIA and the CSA of PVM on MRI; however, due to the small sample size in that study, the accuracy of TMM–BIA could not be adequately investigated. Thus, the purpose of this study was to verify whether TMM–BIA correlates with the quantitative and functional assessments traditionally used for trunk muscles.

## 2. Materials and Methods

### 2.1. Ethics Approval

This study used data obtained from the Shiraniwa Elderly Cohort (Shiraniwa) study [20]. The study protocol was approved by the Institutional Review Board of Osaka City University Graduate School of Medicine (No. 3484). All methods were performed in accordance with the Declaration of Helsinki and the Ethical Guidelines for Medical and Health Research Involving Human Subjects in Japan. Written informed consent was obtained from each participant.

### 2.2. Study Population

**Figure 1:**
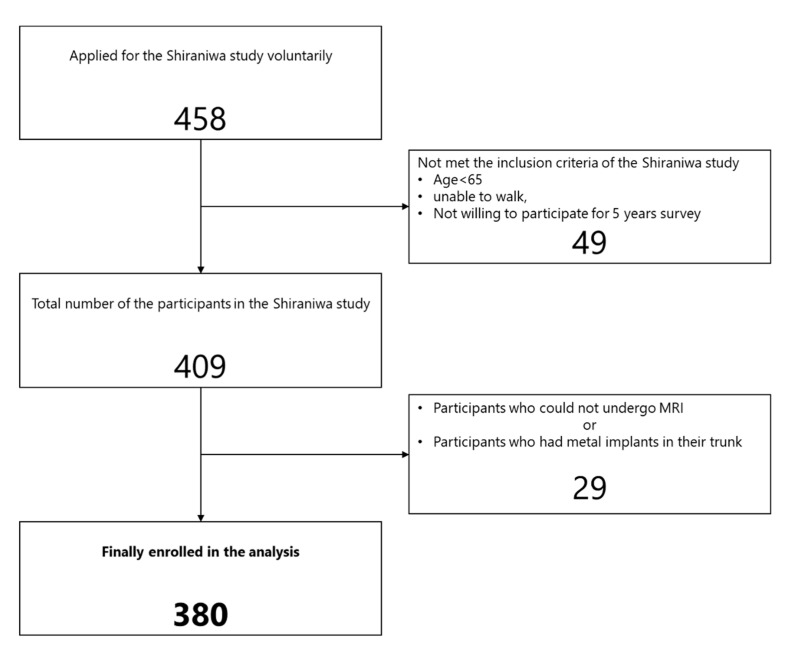
Flowchart of the included and excluded participants. A total of 380 participants were enrolled for this analysis.

The Shiraniwa study is a prospective cohort study that investigates sarcopenia, locomotive syndrome, frailty, and spinal sagittal imbalance among elderly people (aged 65 years or more) living in suburban areas of Japan and recruited by community notices and bulletin boards within our hospital. The inclusion criteria of the subjects were as follows: able to visit the hospital for the survey, able to walk independently, and willing to participate in annual surveys for 5 years. In total, 458 people applied voluntarily and were sent consent forms and self-administered questionnaires. After written consent was obtained, 409 participants (164 males, 245 females; mean age, 73.5 years; SD, 5.4 years) were finally included in the Shiraniwa study. In this analysis, we obtained the data from the first-year survey of the Shiraniwa study and excluded participants who could not undergo MRI or who had metal implants for spinal fusion surgery in their trunk (Figure 1).

### 2.3. Measurements

All of the following measurements were performed on the same day for each participant.

#### 2.3.1. Trunk Muscle Mass Measurement by BIA (TMM–BIA)

We measured the trunk muscle mass (kg) of participants using the BIA method with a body composition analyzer (MC-780A, Tanita Co., Tokyo, Japan). BIA is a non-invasive examination technique used to determine body composition by measuring the electrical resistance (bioimpedance) of living tissues [21]. The BIA device (MC-780A) measures bioimpedance using six electrical frequencies (1, 5, 50, 250, 500, and 1000 kHz). It can accurately identify bone and fat because it distinguishes tissues by their bioimpedance. Muscle mass (kg) was calculated by subtracting fat mass and bone mass from the total body weight (kg). Furthermore, trunk muscle mass (kg) was calculated by subtracting the ASM (kg) from the muscle mass of the whole body (kg).

#### 2.3.2. Quantitative Evaluation of Trunk Muscle on MRI

In this study, MRI evaluations were performed using the Achieva 3.0 Quasar (Koninklijke Philips N.V., Amsterdam, Netherlands). A T2-weighted axial image (TR = 7670, TE = 90, FOV = 170 × 170 mm, slice = 5 mm) was used to measure the CSA of PVM, including ES, MF, and PM, at the L3/4 level, using the “pencil tool” from the 32-bit OsiriX software (version 3.8.1, Pixmeo, Geneva, Switzerland). The CSA including infiltrated fat was measured and determined, and then the intramuscular fat based on regions of interest (ROIs) with intensity changes was differentiated. The fat-free CSA for each PVM was then calculated as the difference between these two values [22].

#### 2.3.3. Functional Evaluation of Trunk Muscles

The BMS of each participant was determined by measuring the maximal isometric strength of the trunk muscles in a standing position with 30° of lumbar flexion using a digital BMS meter (T.K.K.5402, TAKEI, Niigata, Japan) [10,11]. After performing warm-up exercises called “radio calisthenics”, the participants underwent the BMS measurement twice. The average force from two trials was recorded. As the minimum measurable value of the digital BMS meter is 20 kg, in case the participant’s BMS was too weak to be measured, it was not recorded and was excluded from the analysis of BMS.

### 2.4. Statistical Analysis

We investigated the correlation between TMM–BIA and the CSA of PVM and fat-free CSA of PVM using Spearman’s rank correlation coefficient. The relationship between TMM–BIA and the CSA of each individual PVM (ES, MF, and PM) was also evaluated. Additionally, we examined the association between TMM–BIA and BMS. Patient demographics were compared using Student’s *t*-tests. All statistical analyses were performed using Statistical Package for the Social Sciences (SPSS Inc., version 19.0, Chicago, IL, USA). Correlation strengths were categorized as very weak (<0.20), weak (0.20–0.39), moderate (0.40–0.59), strong (0.60–0.79), or very strong (≥0.80). Statistical significance was set at *p* < 0.05.

## 3. Results

Data from 380 participants in the Shiraniwa study (152 males, 228 females; mean age, 73.4 years) who underwent TMM–BIA, lumbar MRI, and BMS measurements were analyzed in this study. The participants’ characteristics are summarized in Table 1.

**Figure 2:**
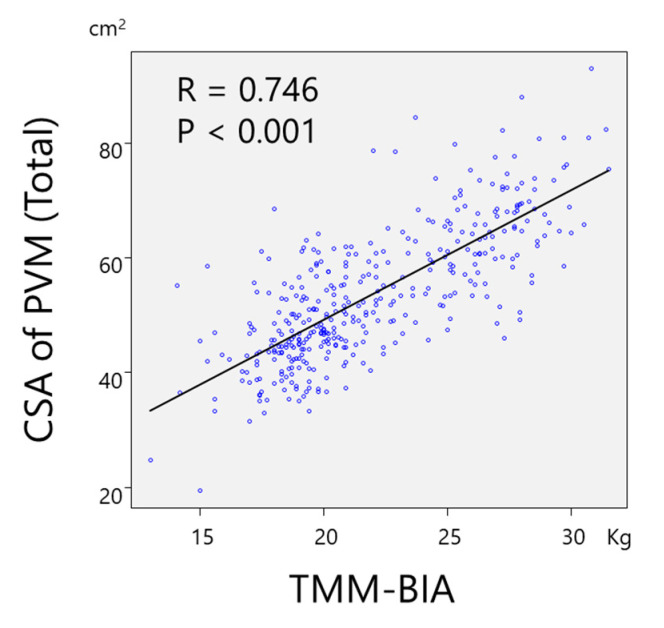
Correlation between TMM–BIA and the CSA of PVM. There was a significant correlation between TMM–BIA and the CSA of PVM with r = 0.746. TMM–BIA, trunk muscle mass measured by bioelectrical impedance analysis; CSA, cross-sectional area; PVM, paravertebral muscles.

**Figure 3:**
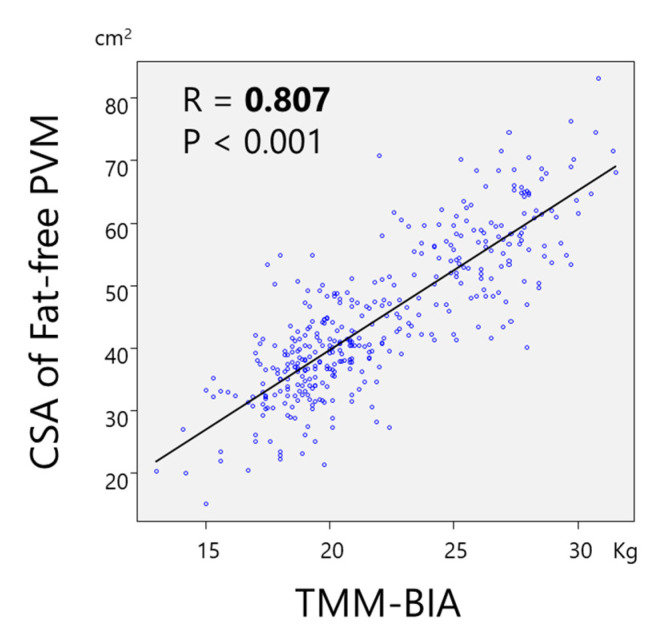
Correlation between TMM–BIA and the CSA of PVM without intramuscular fat. There was a significant correlation between TMM–BIA and the CSA of fat-free PVM with r = 0.807. TMM–BIA, trunk muscle mass measured by bioelectrical impedance analysis; CSA, cross-sectional area; PVM, paravertebral muscles.

**Figure 4:**
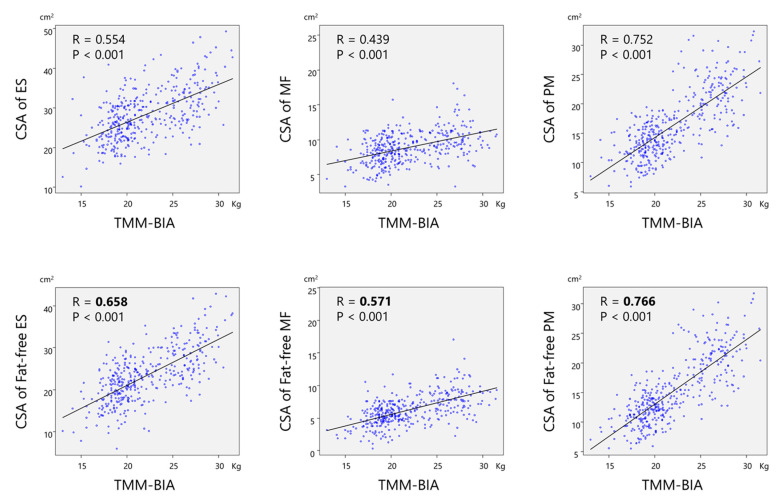
Correlations between TMM–BIA and the CSA of each individual PVM (upper row, total; lower row, excluding intramuscular fat). The CSA of the PM showed a strong correlation with the TMM–BIA (total, r = 0.752; fat-free, r = 0.766), whereas the CSA of MF, the smallest muscle of the PVM, showed a moderate correlation (total, r = 0.439; fat-free, r = 0.571). In addition, the CSA of ES had a moderate to strong correlation to the TMM–BIA (total, r = 0.554; fat-free, r = 0.658), respectively. TMM–BIA, trunk muscle mass measured by bioelectrical impedance analysis; CSA, cross-sectional area; PVM, paravertebral muscle; ES, erector spinae; MF, multifidus; PM, psoas major.

**Figure 5:**
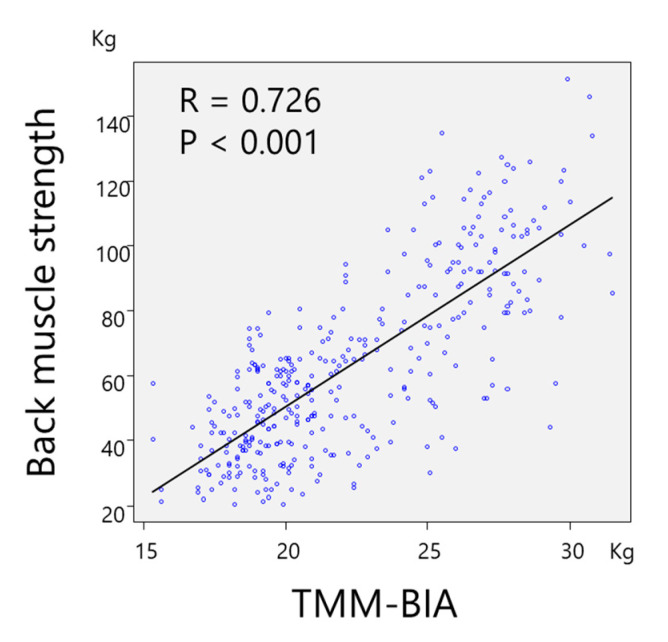
Correlation between TMM–BIA and back muscle strength. There was a strong correlation of r = 0.726, even though some of the participants exhibited minimum strength because of pain. TMM–BIA, trunk muscle mass measured by bioelectrical impedance analysis.

A significant and strong correlation was found between TMM–BIA and the CSA of PVM (r = 0.746, *p* < 0.01) (Figure 2), and between TMM–BIA and the fat-free CSA of PVM (r = 0.807; *p* < 0.01) (Figure 3). Similarly, TMM–BIA was significantly correlated with the CSA of each individual PVM (Figure 4). The CSA of PM was strongly correlated with the TMM–BIA (fat included, r = 0.752, *p* < 0.01; fat-free, r = 0.766, *p* < 0.01), whereas the CSA of MF, the smallest muscle of the PVM, was moderately correlated with TMM–BIA (fat included, r = 0.439, *p* < 0.01; fat-free, r = 0.571, *p* < 0.01). In addition, the CSA of ES was moderately correlated with TMM–BIA (fat included, r = 0.554, *p* < 0.01; fat-free, r = 0.658, *p* < 0.01) (Table 2). TMM–BIA and BMS were strongly correlated (r = 0.726, *p* < 0.001), although the strength of some participants could not be measured due to back pain (Figure 5).

### 3.1. Case Presentation

#### 3.1.1. Case 1

**Figure 6:**
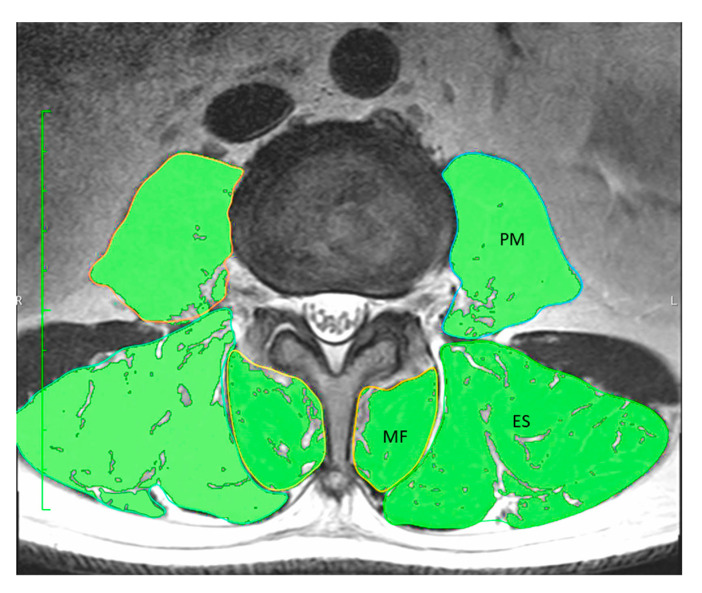
Case presentation 1. A 67-year-old male with no symptoms of low back pain had a CSA of PVM and fat-free PVM of 75.4 and 68.04 cm^2^, respectively. The fat-free percentage of PVM was 90.2%. CSA, cross-sectional area; PVM, paravertebral muscles.

A 67-year-old male with a history of hepatitis and diabetes mellitus had a high TMM–BIA of 31.5 kg. The CSA of PVM on the MRI was 75.4 cm^2^ (fat included) and 68.04 cm^2^ (fat-free) (Figure 6). He reported his low back pain as 0 mm on a visual analog scale. His back muscle strength was 85.5 kg.

#### 3.1.2. Case 2

**Figure 7:**
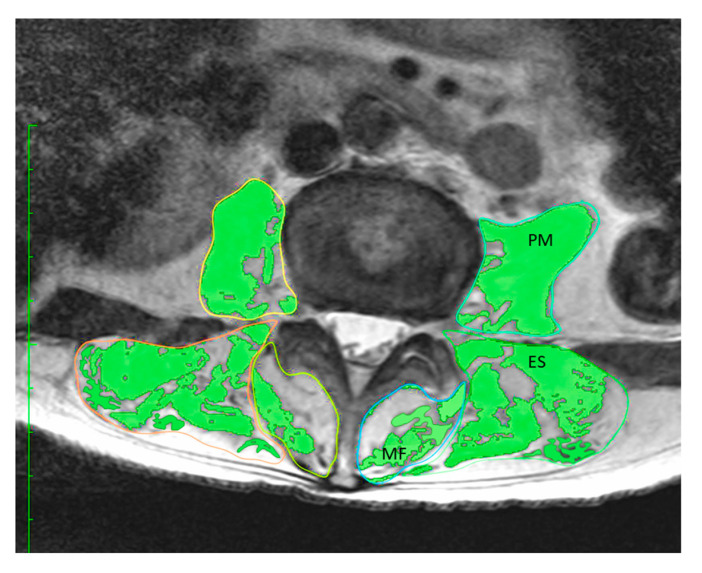
Case presentation 2. A 70-year-old female with severe low back pain. The patient’s CSA of PVM and fat-free PVM was 36.59 and 20.02 cm^2^, respectively. The fat-free percentage of PVM was 54.7%. TMM–BIA, trunk muscle mass measured by bioelectrical impedance analysis; CSA, cross-sectional area; PVM, paravertebral muscles.

A 70-year-old female with a history of diabetes mellitus and osteoporosis had a low TMM–BIA volume of 14.2 kg. MRI showed severe muscular atrophy and fatty degeneration in her PVM (Figure 7). The CSA was 36.59 cm^2^ (fat included) and 20.02 cm^2^ (fat-free). She reported severe low back pain as 76 mm on a visual analog scale. Her back muscle strength was too weak to be recorded (less than 20 kg).

## 4. Discussion

The clinical importance of TMM–BIA was first reported by Hori et al. [12]. They conducted a multicenter, cross-sectional study of 1738 patients (mean age, 70.2 ± 11.0 years; 781 males and 957 females) and found that TMM–BIA was significantly associated with various spinal pathologies, including low back pain, quality of life related to low back pain, and spinal sagittal imbalance, indicating that TMM–BIA is a useful indicator for understanding the pathology of the spine in clinical settings. However, only one study [19] has investigated the association between TMM–BIA and other pre-existing assessment methods for trunk muscles; therefore, the accuracy of TMM–BIA has yet to be validated.

The present study is the first validation study of TMM–BIA, and the results of this study indicate a strong correlation between TMM–BIA and the CSA of PVM. Furthermore, we clarified that TMM–BIA is more strongly correlated with the CSA of PVM, excluding fat infiltration, than the total PVM. Our results suggest that TMM–BIA is a valid index of trunk muscle mass.

The CSA of PVM and fat infiltration of the PVM measured via MRI or CT has been widely used for quantitative evaluation of the trunk muscles. Many studies have sought to investigate the association between the CSA, or fat infiltration, and spinal pathologies [2,23,24]. Takahashi et al. [24] reported that a decrease in PVM in patients with osteoporotic vertebral fractures was significantly related to low back pain and delayed union after fracture onset. Kjaer et al. determined that fat infiltration of the MF was associated with low back pain in adults [25]. Sasaki et al. [26] found that the fatty infiltration ratio of the ES in the upper lumbar spine was significantly associated with low back pain. However, the widespread use of MRI or CT for the evaluation of trunk muscle mass is impractical, as it is time-consuming and expensive, and CT exposes patients to radiation. In contrast, TMM–BIA is a straightforward, non-invasive, and reliable method for large-scale measurements.

Functional assessment of the trunk muscles was performed via BMS. Several studies have reported that BMS may be a useful index for spinal pathology and function, such as spinal sagittal alignment [27], thoracic kyphosis [28], and range of motion of the spine [29]. Despite its clinical importance, the measurement of BMS is difficult in patients with low back pain, and has the potential risk of vertebral fracture in patients with severe osteoporosis [30]. We found a strong correlation between TMM–BIA and BMS using a relatively large sample size, which indicates that TMM–BIA is an accurate tool for the functional assessment of trunk muscles without any risk of adverse effects.

Our study had several limitations. First, TMM–BIA includes the total volume of all trunk muscles (not only PVM); however, we could only measure the CSA of PVM. Therefore, other trunk muscles were not evaluated using TMM–BIA in this study. Second, in this study, we measured trunk muscle mass using only one type of BIA device. It has been reported that the ASM varies depending on the type and manufacturer of the BIA device [31]. Therefore, trunk muscle masses may differ when another BIA device is used. A conversion formula that shows the same ASM across BIA devices has been reported [32]. Future studies to develop a similar conversion formula for trunk muscle mass are needed. Third, we did not analyze the influence of sex or age in this study. There were significant differences in CSA, BMS, and TMM–BIA between male and female study participants (Table 1). As the purpose of this study was first to verify whether TMM–BIA correlates with the quantitative and functional assessments traditionally used for trunk muscles, an examination of the influence of sex or age on the relationship of TMM–BIA and the CSA of PVM will be the subject of our next research work. Last, this study was a cross-sectional analysis of the relationship between TMM–BIA and the CSA of PVM using data collected on the same day. Therefore, the relationship between changes in TMM–BIA and those in the CSA of PVM was not studied. Future studies should focus on analyzing the changes in these parameters via a longitudinal study design.

## 5. Conclusions

TMM–BIA is strongly correlated with the CSA of PVM, especially the fat-free CSA, as measured with MRI. Additionally, TMM–BIA is correlated with BMS. As CSA and BMS are gold standards for quantitative and functional assessments of trunk muscles, TMM–BIA can be considered a new method to measure these parameters. Our findings highlight the significance of TMM–BIA as a reliable, cost-effective, and efficient tool for the assessment of trunk muscles. Given its simplicity and reliability, BIA may be an alternative method for evaluating trunk muscles in clinical settings.

## Figures and Tables

**Table 1:** Characteristics of the participants of the Shiraniwa study.

	Total	Male	Female	*p*-Value
Number of participants	380	152	228	
Age, years.	73.4 (5.3)	73.7 (5.2)	73.3 (5.5)	0.81
Height, cm	156.4 (9.1)	164.9 (5.9)	150.8 (6.0)	<0.01
Weight, kg	56.5 (10.6)	63.7 (8.7)	51.8 (8.9)	<0.01
BMI, kg/m^2^	23.0 (3.3)	23.4 (2.8)	22.7 (3.6)	0.04
Back muscle strength, kg	60.0 (29.2)	84.2 (26.3)	47.3 (15.0)	<0.01
Number of participants whose BMS was too weak to be recorded	20 (5.3%)	1 (0.7%)	19 (8.3%)	<0.01
TMM–BIA, kg	21.95 (3.87)	25.94 (2.55)	19.28 (1.72)	<0.01
CSA of PVM, cm^2^ (Total)	54.54 (11.56)	63.34 (9.40)	46.97 (7.52)	<0.01
CSA of Fat-free PVM, cm^2^ (excluding intramuscular fat)	44.70 (11.95)	55.70 (9.15)	37.36 (6.89)	<0.01
Fat-free percentage of PVM, % (excluding intramuscular fat/Total)	83.0 (9.2)	87.9 (6.4)	79.7 (9.3)	<0.01

Data are presented as mean (standard deviation). BMI, body mass index; TMM–BIA, trunk muscle mass measured by bioelectrical impedance analysis; CSA, cross-sectional area; PVM, paravertebral muscles. Student’s *t*-test was used to compare groups.

**Table 2:** Correlations between TMM–BIA and each PVM with and without intramuscular fat.

	CSA, cm^2^	R with TMM–BIA	*p*-Value
ES	28.26 (6.36)	0.554	<0.01
ES (excluding intramuscular fat)	23.25 (6.22)	0.658	<0.01
MF	8.93 (2.40)	0.439	<0.01
MF (excluding intramuscular fat)	6.27 (2.48)	0.571	<0.01
PM	16.29 (5.38)	0.752	<0.01
PM (excluding intramuscular fat)	15.17 (5.39)	0.766	<0.01
Total PVM	54.54 (11.56)	0.746	<0.01
Total PVM (excluding intramuscular fat)	44.70 (11.95)	0.807	<0.01

Data are presented as mean (standard deviation). TMM–BIA, trunk muscle mass measured by bioelectrical impedance analysis; CSA, cross-sectional area; PVM, paravertebral muscles; R, correlation coefficient; ES, erector spinae; MF, multifidus; PM, psoas major.

## Data Availability

Data available on request due to restrictions e.g. privacy or ethical. The data presented in this study are available on request from the corresponding author. The data are not publicly available due to patients privacy.
